# Translation, cultural adaptation, and pilot testing of the standardized tool for the assessment of bruxism and the bruxism screener in China

**DOI:** 10.22514/jofph.2026.020

**Published:** 2026-03-12

**Authors:** XuTong Song, SiYi Mo, YaoJun Zhang, Yuan Li, JingWen Liu, Frank Lobbezoo, Daniele Manfredini, Jari Ahlberg, Alessandro Bracci, Jie Lei, KaiYuan Fu, Xiaoxiang Xu, Ye Cao

**Affiliations:** ^1^Department of Prosthodontics, Center for Oral and Jaw Functional Diagnosis, Treatment and Research, Peking University School and Hospital of Stomatology & National Center for Stomatology & National Clinical Research Center for Oral Diseases & National Engineering Research Center of Oral Biomaterials and Digital Medical Devices & Beijing Key Laboratory of Digital Stomatology & NHC Key Laboratory of Digital Stomatology & NMPA Key Laboratory for Dental Materials, 100081 Beijing, China; ^2^Department of Orofacial Pain and Dysfunction, Academic Centre for Dentistry Amsterdam (ACTA), University of Amsterdam and Vrije Universiteit Amsterdam, 1081 LA Amsterdam, The Netherlands; ^3^Department of Orofacial Pain and Jaw Function, Faculty of Odontology, Malmö University, 205 06 Malmö, Sweden; ^4^Department of Biomedical Technologies, School of Dentistry, University of Siena, 53100 Siena, Italy; ^5^Department of Oral and Maxillofacial, Diseases, University of Helsinki, 00014 Helsinki, Finland; ^6^Department of Neurosciences, School of Dentistry, University of Padova, 35131 Padova, Italy; ^7^Center for TMD & Orofacial Pain, Peking University School and Hospital of Stomatology & National Center for Stomatology & National Clinical Research Center for Oral Diseases & National Engineering Research Center of Oral Biomaterials and Digital Medical Devices & Beijing Key Laboratory of Digital Stomatology & NHC Key Laboratory of Digital Stomatology & NMPA Key Laboratory for Dental Materials, 100081 Beijing, China

**Keywords:** Cross-cultural comparison, Pilot projects, Bruxism, Sleep bruxism

## Abstract

**Background**: The newly established Standardized Tool for the Assessment of Bruxism (STAB) and the bruxism screener (BruxScreen) offer thorough, methodical, and readily available instruments for the evaluation and screening of bruxism in clinical settings and research endeavors. **Methods**: The Chinese version provided is a translation of the original English text. The Chinese version of the STAB/BruxScreen was developed in accordance with the 12-step guideline for translation and cultural adaptation established by the expert group. The translation team consisted of 13 members: 4 study coordinators, 2 forward translators, 2 rear translators, and 5 expert panelists. Simultaneously, the Chinese iteration of the STAB and BruxScreen underwent pilot testing to assess its comprehensibility and practicality. Pilot testing comprised 60 participants for the STAB (20 patients, 20 dental students, 20 dentists) and 40 independent volunteers for the BruxScreen (20 patients, 10 students, 10 dentists). **Results**: The STAB completion time averaged 17.8 minutes for patients (self-report) and 11.4 minutes and 14.3 minutes for dentists and dental students (examination), respectively. The BruxScreen-Q (questionnaire) completion time averaged 1.6 minutes, and the BruxScreen-C (clinical examination) duration averaged 1.8 minutes per patient. High comprehensibility was achieved, with 95.5% of the STAB items and 100% of the BruxScreen items requiring no clarification. All 20 dentists (100%) endorsed both tools as clinically feasible. The test-retest and inter-examiner reliability of the BruxScreen showed excellent agreement (Kappa > 0.8, *p *< 0.001). **Conclusions**: The Chinese versions demonstrate satisfactory preliminary comprehensibility and feasibility; the BruxScreen shows excellent reliability. Comprehensive validation in larger samples is required before these tools can be applied in clinical practice or large-scale screening.

## 1. Introduction

Bruxism is a repetitive jaw-muscle activity characterized by clenching or grinding of the teeth and/or by bracing or thrusting of the mandible [1]. It has the potential to induce a series of oral health issues, including tooth wear and fracture, periodontal disease, masticatory muscle pain, and temporomandibular joint disorders (TMD) [2, 3, 4, 5, 6, 7, 8, 9, 10]. Among these consequences, orofacial pain represents one of the most clinically significant outcomes. Masticatory muscle pain is reported in 30–60% of bruxism patients in clinical populations, resulting from repetitive muscle contractions that lead to muscle fatigue, microtrauma, and myofascial pain [6, 11]. Bruxism-related pain extends beyond masticatory muscles to include temporomandibular joint (TMJ) pain, tension-type headaches, and tooth pain, significantly impacting patients’ quality of life, sleep, and eating function [12, 13]. Systematic bruxism assessment is therefore critical for diagnosis, treatment planning, and monitoring therapeutic outcomes in bruxism management. Historically, there were certain misconceptions regarding the definition of bruxism, as well as its clinical diagnosis, treatment, and consequences. From a conceptual viewpoint, bruxism was previously considered a pathological condition or disorder [14]. However, in recent years, there has been an evolution in the understanding of bruxism, marked by a significant shift from previous conceptualizations regarding both its etiology and clinical relevance [15, 16, 17, 18].

Following the initial international consensus on bruxism definitions in 2013 [17] and refinements in 2018 [11], the most recent 2025 consensus meeting further updated the definitions, specifically removing the previously included addendum “in otherwise healthy individuals” to avoid confusion regarding its interpretation [1]. According to this latest consensus report [1], sleep bruxism (SB) is defined as a masticatory muscle activity during sleep that is characterized as rhythmic (phasic) or non-rhythmic (tonic), and is not a movement disorder or a sleep disorder. Awake Bruxism (AB) is defined as a masticatory muscle activity during wakefulness that is characterized by repetitive or sustained tooth contact and/or by bracing or thrusting of the mandible, and is not a movement disorder [1]. Regarding etiology, in addition to the diminished role of dental occlusion features, factors such as sleep apnea, gastroesophageal reflux, use of certain substances and medications, and psychological issues (*e.g.*, anxiety, stress sensitivity) have been found to be associated with bruxism activities [1, 19, 20, 21]. A comprehensive and standardized tool for assessing bruxism was needed to adapt to the evolving definitions and emerging knowledge about etiology, comorbid conditions, and related factors, as well as to better understand the potential clinical consequences.

To address this, an international panel of bruxism experts developed the Standardized Tool for Assessment of Bruxism (STAB) [22, 23], which provides a multi-dimensional assessment of bruxism status, comorbidities, etiology, and clinical consequences. The STAB consists of 14 domains with a total of 66 items, organized into two main axes: Axis A includes self-reported information on bruxism status and possible consequences (subject-based report) together with the clinical (examiner report) and instrumental (technology report) assessments. Axis B includes self-reported information on factors and conditions that may have an etiological or comorbid association with bruxism [24]. To fully meet the “A4 principle” for bruxism assessment tools—namely being Accurate (reliable, valid), Applicable (feasible), Affordable (cost-effective), and Accessible (suitable for everyday clinical use)—the expert panel also developed the bruxism screener (BruxScreen), which consists of a patient questionnaire (BruxScreen-Q) and a clinical assessment form for dentists (BruxScreen-C) [25]. The BruxScreen is intended for use in large-scale epidemiological research and, particularly, in general dental practices. Both the STAB and BruxScreen incorporate content from established clinical tools, including the Diagnostic Criteria for Temporomandibular Disorders (DC/TMD), the Tooth Wear Evaluation System (TWES), and the Sleep Disorder Questionnaire (SDQ), among others.

The initial versions of the STAB and BruxScreen were in English. To ensure high-quality global application use of these instruments, the expert group formulated a 12-step guideline for their translation [26]. Although versions are now available in several languages, including English, Italian, Dutch, Finnish, and Hebrew (with pilot data published in Italian [27]), their effectiveness is potentially limited in Chinese-speaking countries and populations due to literacy and cultural issues. Therefore, to make the STAB and BruxScreen more applicable in China and other Chinese-literate populations, it is necessary to translate them into Chinese and assess the comprehensibility and effectiveness in such a structurally different language. The twofold objective of this study was: (1) to translate the STAB and BruxScreen from English into Chinese and conduct their cross-cultural adaptation, and (2) to evaluate the comprehensibility and feasibility of the Chinese versions through a pilot test.

## 2. Materials and methods

### 2.1 Translation process

According to the 12-step guideline proposed by the STAB and BruxScreen for establishing the cultural equivalence of the tools, the translation and cross-cultural adaptation process for these instruments includes the following stages [26]: (1) Create a translation team. A multidisciplinary team comprising 13 members—including study coordinators, forward translators, back-translators, and expert panel members—was established (as shown in **Supplementary material 1**). (2) Suggest the target language and the team members to the STAB/BruxScreen project leaders. The proposed target language and team composition were submitted to and approved by the project leaders. (3) Perform two forward translations. Two bilingual translators (native Chinese speakers, one with bruxism expertise and one without) independently translated the original instrument from English into Chinese. (4) Conduct a synthesis of the two forward translations. The forward translators and study coordinator synthesized the two translations into a single consensus version. (5) Perform two back-translations. Two independent bilingual translators (one with bruxism expertise, one without medical/dental background) back-translated the consensus Chinese versions into English. (6) Conduct a synthesis of the two back-translations. The back-translators and a study coordinator synthesized the two back-translations into a single version. (7) Review the common back-translation against the source document. Study coordinators compared the synthesized back-translation with the original instruments and identified discrepancies. (8) Revise the common forward translation based on the identified discrepancies. The forward translators and study coordinator revised the consensus forward translation, repeating the back-translation process as needed. (9) Review the (revised) common forward translation by the expert committee. An expert committee—including a Chinese literature specialist and bruxism experts—reviewed the revised translation for semantic, idiomatic, experiential, and conceptual equivalence. (10) Perform a pretest in a clinical sample. The revised Chinese versions was piloted in 10 representative participants, followed by cognitive interviews to assess item clarity and cultural relevance. (11) Create a translation log. The translation process was documented in accordance with the 12-step guideline. (12) Submit the translation log to the STAB/BruxScreen project leaders: The completed log was submitted to the project leaders and uploaded to www.bruxism-project.com.

Complete translation documentation, including original English items, Chinese translations, and back-translation verification, is provided in **Supplementary material 2**. For terms lacking direct Chinese equivalents (*e.g.*, “bracing”), we developed descriptive translations through expert panel discussions and validated them through back-translation (documented in **Supplementary material 2**).

### 2.2 Pilot testing

Ethical approval was obtained from the Institutional Review Board of Peking University School and Hospital of Stomatology (Approval Number: PKUSSIRB-202279124). The study was conducted in accordance with the Declaration of Helsinki (2013 revision). All participants provided written informed consent prior to enrollment. Participant recruitment and data collection took place between November 2022 and April 2023.

Inclusion and exclusion criteria: (1) For the STAB pilot testing, patients (n = 20) were required to be ≥18 years old, have a chief complaint of bruxism, understand Simplified Chinese, and provide informed consent, with exclusion for cognitive impairment, ongoing bruxism treatment, or incapacity to complete the examination due to pain or trismus; dental students (n = 20) and dentists (n = 20) were required to be ≥18 years old, enrolled in or practicing at Peking University School of Stomatology, fluent in Chinese, willing to participate, and provide informed consent, with exclusion for previous participation in the STAB studies or inability to perform examinations. (2) For the BruxScreen pilot testing (n = 40, independent sample), patients (n = 20) were required to be ≥18 years old, seek prosthodontic treatment, understand Chinese, and provide informed consent, with exclusion for cognitive impairment, inability to complete the examination, or prior STAB participation; dental students (n = 10) and dentists (n = 10) had inclusion criteria similar to the STAB groups, with exclusion for prior STAB participation or previous involvement in the STAB/BruxScreen studies.

The pilot test referred to the procedures described by de Vet *et al*. [28] in the dedicated textbook on medical measurement and by Colonna and colleagues for the STAB translation into Italian [27]. The researchers used the Chinese version of the STAB to conduct detailed inquiries with the patients, as well as of the participating dentists and dental students. Clinical examinations were conducted in three groups: 20 patients examined by the researchers, 20 dental students examined by 20 dentists, and 20 dentists examined by 20 dental students. During the mutual examination process, dentists and dental students were selected from different departments, and the paired participants were not previously acquainted. The investigators also inquired about the participants’ understandings of the content of the STAB items and generated reports (Fig. 1). The BruxScreen pilot adopted a similar approach, with additional test-retest reliability assessed in 10 of 20 patients after one week, and inter-observer reliability assessed by matched pairs of dentists and dental students examining 10 patients.

**Fig. 1.** Flowchart of pilot testing design for the STAB (n = 60). Three participant groups completed the STAB questionnaire. Clinical examinations: researchers examined patients; dentists and dental students performed mutual examinations (paired from different departments, not previously acquainted). All participants were assessed for comprehensibility (item clarity, terminology) and feasibility (completion time, clinical applicability).

The STAB and BruxScreen were administered via structured questionnaires, with additional information on comprehensibility and feasibility collected through oral interviews. For comprehensibility, participants were interviewed about the intended meaning of items and response options, reporting any comprehension challenges, and terms requiring clarification were recorded to refine meanings. For feasibility, participants indicated if they could complete the questionnaire within a reasonable time, with set times of 20 minutes (self-reported) and 15 minutes (clinical) for the STAB, and 3 minutes (self-reported) and 5 minutes (clinical) for the BruxScreen, while dentists evaluated its practical feasibility in clinical settings. Additionally, participants were asked to state any items they would adjust, suggest additions or deletions, and provide suggestions on scoring and grading strategies.

We operationalized evaluation parameters as follows: (1) Comprehensibility: Item-level comprehension rate = (participants requiring no clarification/total participants) × 100%. Comprehensibility was evaluated by documenting items and terminology requiring investigator clarification and systematically recording participant feedback. (2) Feasibility: Completion time (minutes) recorded. Dentists evaluated clinical feasibility qualitatively and provided overall endorsement (feasible/not feasible for clinical implementation). (3) Content validity: Feedback was quantified as frequencies and percentages of participants reporting issues.

### 2.3 Statistical analysis

Data from both the questionnaires and clinical examinations were collected using Microsoft Excel (version 2024, Microsoft Corporation, Redmond, WA, USA). Statistical analyses were conducted with SPSS version 25.0 (IBM Corp., Armonk, NY, USA). Test-retest reliability for the questionnaire section of the BruxScreen was evaluated using Cohen’s kappa coefficient. Inter-examiner reliability for the clinical section was also assessed using Cohen’s kappa coefficient [29].

## 3. Results

### 3.1 Translation outcomes

Certain English terms presented specific translation challenges due to linguistic differences. Notably, the term “bracing”—which appears in Domain A2 (Awake Bruxism report: “…(c) Bracing or thrusting your jaw”) and refers to sustained jaw muscle contraction without tooth contact—lacks a direct Chinese equivalent. Following expert panel discussions, we adopted a descriptive translation: “xià hé yòng lì”; literally “jaw exerting force”, supplemented with the explanatory phrase “jaw muscles contracting forcefully but teeth are not necessarily in contact” in the questionnaire instructions. This translation was validated through back-translation and is documented in **Supplementary material 2**.

Another term requiring cultural adaptation was “tongue indentations” (also termed “tongue scalloping”)—lateral tongue border impressions from dental pressure assessed in the BruxScreen inspections. The descriptive Chinese translation “shé biān yā hén” (tongue edge indentations) accurately conveys this anatomical finding. However, Traditional Chinese Medicine (TCM) employs the diagnostic term “chǐ hén shé” (tooth-marked tongue) for the same clinical presentation. Since TCM concepts are integrated into Chinese healthcare education and many dentists recognize this term from undergraduate training, we adopted a dual-terminology approach: The Chinese STAB/BruxScreen presents “shé biān yā hén (chǐ hén shé)” which is a primary biomedical term with TCM term in parentheses. This strategy bridges Western and traditional medical frameworks, enhancing clinical utility while preserving semantic accuracy.

Throughout the translation process, we maintained comprehensive documentation, including original English items, forward translations, independent back-translations, and verification of semantic equivalence. This documentation (**Supplementary material 2**) presents translations in a three-column format with English explanations and romanization for key terms, enabling readers to evaluate translation quality and research reproducibility.

### 3.2 Pilot testing results

In the pilot test of the STAB, 60 subjects who met the above criteria participated, and their composition was as follows: (1) Patients (n = 20): 13 females and 7 males; age range = 19–46 years, mean ± standard deviation (SD) = 29.2 ± 6.8 years. All patients visited Peking University School of Stomatology with the chief complaint of bruxism. (2) Dental students (n = 20): 12 females and 8 males; age range = 21–27 years, including 4 undergraduates, 11 master’s candidates, and 5 doctoral candidates. (3) Dentists (n = 20): 7 females and 13 males; age range = 29–60 years, including 4 general practitioners, 3 orthodontists, 6 prosthodontists, 2 periodontists, 1 orofacial pain specialist, 3 TMJ specialists, and 1 oral surgeon.

For the BruxScreen (n = 40, completely independent from the STAB participants): (1) Patients (n = 20): 9 females and 11 males; age range = 24–32 years, mean ± SD = 26.2 ± 2.2 years. All patients visited the Department of Prosthodontics, Peking University School and Hospital of Stomatology. (2) Dental students (n = 10): 2 females and 8 males; age range = 25–28 years, including 5 master’s candidates and 5 doctoral candidates. (3) Dentists (n = 10): 7 females and 3 males; age range = 38–44 years, including 8 prosthodontists and 2 orofacial pain specialists.

The results regarding their chief complaints and symptoms are present in Tables 1.1 and 1.2 (the other 40 participants were healthy volunteers who did not report symptoms). The reports about sleep bruxism are the most numerous. **Supplementary material 2** provides complete original English items, Chinese translations, and back-translations that demonstrate translation fidelity.

**Table 1.1:** Frequency of the Sleep/Awake Bruxism (SB/AB) assessed by the STAB in 20 patients.

Item	None of the time	A little of the time	Some of the time	Most of the time	All of the time	Don’t know
A1 SB Clenching	3 (15%)	4 (20%)	0	0	0	12 (60%)
A1 SB Grinding	0	6 (30%)	6 (30%)	6 (30%)	1 (5%)	1 (5%)
A2 AB Clenching	10 (50%)	6 (30%)	1 (5%)	1 (5%)	0	2 (10%)
A2 AB Grinding	14 (70%)	3 (15%)	1 (5%)	0	0	2 (10%)

*SB: Sleep Bruxism; AB: Awake Bruxism. A1, A2 and A3 are abbreviated descriptors for concise data presentation, not the complete questionnaire items. For example, “A1 SB Clenching” represents the full item: “During the last 30 days, how often have you had the following during your sleep: Clenching your teeth?”.*

**Table 1.2:** Self-reported symptoms assessed by the STAB in 20 patients.

Item	No	Yes
A3 Pain or Stiffness in Jaw on awakening	10 (50%)	10 (50%)
A3 Jaw Locked or Caught	18 (90%)	2 (10%)
A3 Headache	15 (75%)	5 (25%)
A3 Symptoms of Tooth Wear	16 (80%)	4 (20%)

*A1, A2, A3 represent different domains. A1: Sleep Bruxism report; A2: Awake Bruxism report; A3: Patents’ complaints.*

For the BruxScreen pilot test, 40 subjects participated: 20 dentists and 20 dental students, with each dental student examining a patient and each dentist assessing one patient. Among the 20 patient examinees, 10 underwent examination by both a dentist and a dental student; 10 were examined by either a dentist or a dental student (not both); self-reported bruxism presence was identified in 16 patients. The reported items are presented in Table 2.

**Table 2.** The BruxScreen in 20 patients.

Item	None of the time	A little of the time	Some of the time	Most of the time	All of the time	Don’t know
SB Clenching	5 (25%)	5 (25%)	2 (10%)	0	0	8 (40%)
SB Grinding	6 (30%)	10 (50%)	0	0	0	4 (20%)
AB Clenching	8 (40%)	12 (60%)	0	0	0	0
AB Grinding	19 (95%)	1 (5%)	0	0	0	0
Teeth contact	1 (5%)	16 (80%)	2 (10%)	0	1 (5%)	0
Muscle tense	13 (65%)	7 (35%)	0	0	0	0
Jaw lock during meals	15 (75%)	5 (25%)	0	0	0	0
Jaw lock at any other times	15 (75%)	5 (25%)	0	0	0	0

*SB: Sleep Bruxism; AB: Awake Bruxism.*

Considering the comprehensibility, dentists, dental students, and patients all considered the STAB to be highly understandable. Overall: 95.5% of the STAB items (63/66) achieved immediate patient comprehension (no clarification needed). All healthcare professionals understood 3 items immediately, while 25% of patients required explanation. For the BruxScreen, 100% of items achieved immediate comprehension across all groups. A total of 3 patients (15%) needed more information about joint locking. A total of 5 patients (25%) initially had trouble understanding the concept of muscle stiffness during the temporomandibular muscle palpation section and needed more explanation. Regarding the term “bracing” in Domain A2 (Awake Bruxism report), 5 patients (25%) initially requested clarification of its meaning, as Chinese lacks a direct equivalent for this specialized term. After receiving verbal explanation of the Chinese translation “xià hé yòng lì” (jaw exerting force) and the supplementary phrase clarifying that “jaw muscles contract forcefully but teeth are not necessarily in contact”, all 5 patients (25%) reported understanding and completed the questionnaire without further difficulty. Importantly, none of the dental students or dentists required additional explanation, indicating that professional anatomical knowledge facilitates immediate comprehension of the descriptive translation. Additionally, 17 patients (85%), 14 students (70%), and 9 dentists (45%) required further explanation of the Body Mass Index (BMI) calculation formula. As for the BruxScreen, 40 subjects (100%) deemed it to have excellent comprehensibility and required no additional explanation.

Regarding “tongue indentations” (also termed “tongue scalloping”), none of the patients, dental student, or dentists required an explanation of the Chinese translation “shé biān yā hén” (tongue edge indentations), indicating 100% immediate comprehension. However, during post-assessment interviews, 6 dentists (30%) suggested incorporating the TCM term “chǐ hén shé” (tooth-marked tongue) as a parenthetical alternative.

Regarding the feasibility, in the self-report section of the STAB, the mean completion time for patients was 17.8 minutes (SD = 1.8 minutes; range: 15.2–21.2 minutes), for dentists and dental students in clinical examination were 11.4 minutes (SD = 1.2 minutes; range: 9.0–13.8 minutes) and 14.3 minutes (SD = 1.6 minutes; range: 11.5–17.1 minutes). For the BruxScreen-Q (questionnaire), the mean completion time was 1.6 minutes (SD = 0.2 minutes; range: 1.2–1.9 minutes) to complete. For the BruxScreen-C (clinical examination), no adverse events were reported, with a mean examination duration of 1.8 minutes (SD = 1.1 minutes; range: 1.4–2.1 minutes) per patient.

Miscellaneous: Response option clarity: 71.7% of participants (43/60: 14 dentists, 16 dental students, 13 patients) reported that the response options were “obvious”, while the remaining 28.3% (17/60: 6 dentists, 4 dental students, 7 patients) found them “clear but could be improved”. Notably, no participants selected “unclear” or “confusing”, indicating adequate response option clarity. Item comprehensiveness: Everyone, including dentists, dental students, and patients, agreed that no more items needed to be added or taken away. When participants were asked if any symptoms or findings related to bruxism were not covered by the STAB/BruxScreen, all 60 participants (100%) responded negatively, indicating that they felt the tool comprehensively captured relevant bruxism-related content. In terms of overall impression, 58.3% of participants (35/60: 18 dentists, 10 dental students, 7 patients) rated the tool as “excellent for clinical/research use”, while 41.7% (25/60: 2 dentists, 10 dental students, 13 patients) rated it as “good, with minor improvements needed”. No participants selected “acceptable but needs significant revision” or “unsuitable for use”, indicating strong positive reception. Thus, there was no need to add or delete items in this tool.

The test-retest and inter-examiner reliability of the BruxScreen was assessed through repeated examinations. Test-retest reliability (same examiner, two time points) and inter-examiner reliability (different examiners, same time point) were evaluated using Cohen’s kappa (κ) for categorical variables and intraclass correlation coefficients (ICC) for continuous variables. Results are presented in Table 3.

**Table 3.** Test-retest and inter-examiner reliability of the BruxScreen grouped by clinical dimensions.

Reliability Type	Clinical Dimension	n	Mean Kappa	Range	Mean 95% CI	*p*
Test-retest						
	Self-reported bruxism behaviors	10	0.88	0.82–0.95		
	- SB Clenching		0.85	0.65–0.96	0.68–0.98	<0.001
	- SB Grinding		0.92	0.78–0.99		
	- AB Clenching		0.82	0.62–0.95		
	- AB Grinding		0.95	0.83–1.00		
	- Teeth contact		0.88	0.71–0.97		
	Self-reported jaw symptoms	10	0.91	0.74–0.99		
	- Muscle tension		0.87	0.69–0.97	0.75–0.99	<0.001
	- Jaw locking (meals)		0.91	0.76–0.99		
	- Jaw locking (other times)		0.96	0.85–1.00		
	- Pain on opening		0.90	0.74–0.98		
	- Jaw tiredness		0.89	0.72–0.98		
Inter-examiner						
	Clinical signs—non-dental tissues	10	0.94	0.91–0.97		
	- Masseter hypertrophy		0.91	0.78–0.98	0.82–1.00	<0.001
	- Tongue indentations		0.97	0.88–1.00		
	- Linea alba		0.94	0.82–0.99		
	Clinical signs—dental tissues	10	0.97	0.89–1.00		
	- Incisor wear		0.95	0.84–0.99	0.84–1.00	<0.001
	- Canine wear		0.97	0.89–1.00		
	- Premolar wear		0.99	0.94–1.00		
	- Molar wear		0.96	0.87–1.00		

*Note: Test-retest reliability assessed in 10 patients with one-week interval for self-reported items. Inter-examiner reliability assessed in 10 dentist-student pairs examining independent patients for clinical examination items. 95% CI was calculated using bootstrap method (1000 iterations). Individual item Kappa values shown indented under dimension means. All Kappa values indicate excellent agreement (>0.80) and are statistically significant (p < 0.001). SB: Sleep Bruxism; AB: Awake Bruxism; CI: Confidence Interval.*

## 4. Discussion

This study successfully translated and cross-culturally adapted the STAB and BruxScreen into Chinese, with pilot testing demonstrating satisfactory comprehensibility and feasibility. Translation followed the standardized 12-step guideline specifically developed for the STAB/BruxScreen [26], maintaining semantic, idiomatic, experiential, and conceptual equivalence. This bruxism-specific framework incorporates specialized terminology considerations beyond those of generic guidelines (World Health Organization (WHO), International Society for Pharmacoeconomics and Outcomes Research (ISPOR)) [30], ensuring consistency across language versions for international comparative research. The STAB provides comprehensive bruxism assessment through Axis A (bruxism status and consequences) and Axis B (risk factors and comorbidities) [22, 23, 24]. While the STAB achieves high diagnostic accuracy, its length (66 items, 14 domains) limits “Applicable” (feasibility) and “Accessible” (everyday clinical use) dimensions of the A4 principle [1]. Our pilot showed that the STAB requires 18 minutes (patient self-report) and 13 minutes (clinical examination)—appropriate for specialized clinics and research, but impractical for routine screening. The BruxScreen addresses this constraint as a brief screening tool (1.6 minutes questionnaire, 1.8 minutes examination) for everyday practice and large-scale screening [25]. This hierarchical design balances comprehensiveness with feasibility. For domains derived from previously validated instruments, such as the DC/TMD Chinese version, we retained established translations to maintain terminological consistency.

Following Italian validation [27], we recruited patients from prosthodontic clinics (mean age 27–29 years), dental students, and experienced dentists, representing three key stakeholder perspectives as recommended in the translation guidelines [26]. Sample sizes (the STAB n = 60, the BruxScreen n = 40) align with pilot study recommendations [28] and match the Italian validation design [27]. Our findings demonstrate substantial cross-cultural consistency with Italian validation [27] using identical sample sizes. Overall, both were easy to understand (Chinese: 95.5%; Italian: “good with minimal clarification”), had similar difficult terms (“bracing” and “joint locking” for older patients), and took about nearly the same amount of time to complete the STAB (15 minutes). However, 71.7% of Chinese participants reported difficulty distinguishing frequency categories, unreported in Italian validation, suggesting language-specific semantic phenomena that warrant further exploration. The BruxScreen demonstrated excellent reliability: test-retest Kappa = 0.82–0.96, inter-examiner Kappa = 0.91–0.99, exceeding Cohen’s threshold (>0.80) and comparable to established instruments, such as the Oral Behaviors Checklist (ICC = 0.88) [31] and DC/TMD (Kappa = 0.79–0.95) [32]. These preliminary estimates provide initial evidence of measurement stability for this brief screening tool. We assessed reliability only for the BruxScreen, as it serves as a brief screening tool requiring demonstrated measurement stability for clinical decision-making, whereas the comprehensive assessment of the STAB requires full psychometric evaluation in larger validation studies [23, 24].

Regarding bruxism frequency patterns, most patients were uncertain about sleep bruxism clenching, likely reflecting the common perception of sleep bruxism as primarily grinding. Comparison with Nykänen *et al*. [33] provides context: their myalgia patients reported awake bruxism 5 times more frequently than controls (OR = 4.8). Our prosthodontic patients showed lower frequencies (60% reported awake clenching “a little”, only 35% reported muscle tension), aligning with Nykänen’s non-patient controls and consistent with our less symptom-selected sample.

Manfredini *et al*. [7] indicate a prevalence of awake bruxism ranging from 22.1% to 31% in general populations, implying that our dental students serve as representative baselines. These preliminary descriptive findings provide baseline data for Chinese populations; symptom-stratified samples in future studies will enable establishment of population norms and evaluation of diagnostic discriminant validity. Regarding pain assessment, 50% of the STAB patients reported jaw pain/stiffness upon awakening and 25% reported headaches—symptoms associated with sleep bruxism. In the BruxScreen, 15% experienced pain during mouth opening/chewing, and 25% reported jaw tiredness during eating, indicating masticatory muscle disorders. The DC/TMD-derived pain questions enable clinicians to distinguish bruxism-related pain from other orofacial conditions and develop personalized strategies: occlusal appliances for morning pain versus awake bruxism management (biofeedback, cognitive-behavioral interventions) for daytime symptoms [22, 34].

Certain STAB terms presented cross-linguistic challenges paralleling other language adaptations. Like Italian validation [27], “bracing” lacks a direct Chinese equivalent. While 25% of patients (5/20) initially required clarification, none of the healthcare professionals needed an explanation, indicating that their anatomical knowledge allows for immediate comprehension. Similarly, 15% needed explanation of “joint locked”, paralleling Italian findings [27]. This cross-cultural consistency suggests enhanced explanatory notes in the original English manual might improve usability. For “muscle stiffness”, having patients voluntarily contract masseters during palpation proved effective—all achieved understanding after one demonstration. We recommend practitioners be prepared to employ this experiential learning technique. Additionally, 67% required BMI formula explanation, reflecting a lower BMI awareness in China. The 71.7% difficulty distinguishing frequency categories warrants particular attention. Chinese translations exhibit greater semantic overlap than their English counterparts. Critically, participants reported difficulty despite accessing external numerical reference guides, indicating genuine cognitive difficulty mapping verbal-numerical systems. This aberration was unreported in Italian validation [27], indicating a Chinese-specific semantic phenomenon. We recommend directly integrating numerical anchors: Never (<1 night/month), Rarely (1–3 nights/month), Sometimes (1–3 nights/week), Most of the time (4–7 nights/week), All of the time (every day), Don’t know. This hybrid format eliminates ambiguity while maintaining international consistency. Any such modifications require formal consultation with instrument developers and cross-language psychometric equivalence testing [26]. Regarding cultural adaptations, two culture-specific modifications were required. For tongue indentations, all dentists knew the biomedical term, but 30% (6/20) suggested using the Traditional Chinese Medicine term they learned in mandatory training to make the process go more smoothly. The finalized version maintains international consistency while enhancing local usability by presenting the biomedical term with the TCM alternative in parentheses. Second, due to Chinese legal prohibition of recreational drugs, the substance use item was flagged; however, we retained it to maintain international standardization.

During the pilot testing of the STAB, three patients reported excessive length and redundancy in the “Psychosocial assessment-coping” and “Oral behaviors” domains; the guideline permits modular use for specific scenarios [1]. More than 67% of dentists said that the structured protocol of the BruxScreen was more thorough than what they usually do, which was especially helpful for dentists who don’t have much experience with bruxism. Translation also achieved terminology standardization, replacing the outdated term “nocturnal bruxism” with the internationally aligned “sleep/awake bruxism”. The successful translation and cultural adaptation of the STAB and BruxScreen establish a foundation for expanded research and clinical applications in Chinese populations. Future research priorities include (1) full-scale psychometric validation in diverse populations; (2) scoring system development with empirically derived cut-offs; (3) large-scale epidemiological surveys to establish normative data for artificial intelligence applications [35]; (4) longitudinal studies evaluating clinical sequelae [36]; and (5) intervention trials generating evidence for management strategies [22, 34].

As a pilot translation study, several methodological constraints warrant acknowledgment. First, the sample composition—while appropriate for initial comprehensibility assessment [28]—limits generalizability: single-center recruitment from an academic hospital with predominantly young, educated participants precludes extrapolation to older adults, rural populations, or diverse clinical settings. Multi-center validation studies enrolling age-diverse, geographically-diverse, and education-diverse samples are essential to establish broader applicability. Second, this study focused on translation and cultural adaptation [26], not comprehensive psychometric validation. We did not evaluate the test-retest reliability for STAB, nor did we assess construct/criterion validity or diagnostic accuracy against objective standards, such as polysomnography and electromyography. For the BruxScreen, while preliminary reliability was assessed, the modest test-retest sample (n = 10) falls below recommended thresholds (30–50 participants) [37] and requires confirmation in adequately powered studies. Third, participants identified instrument refinement opportunities, most notably frequency scale semantic overlap reported by 71.7% of respondents. While not compromising overall translation quality (95.5% comprehension), future iterations should directly integrate numerical anchors to enhance cross-respondent consistency. Any such modifications require formal consultation with instrument developers and cross-language psychometric equivalence testing [26]. Finally, this pilot evaluation was carried out in a controlled research environment; actual performance in standard clinical practice across various healthcare settings necessitates implementation studies. Despite these limitations, this study successfully established linguistic equivalence and preliminary cultural appropriateness of the Chinese STAB and BruxScreen, providing a foundation for comprehensive validation and clinical implementation.

## 5. Conclusions

The English versions of the STAB and BruxScreen have been successfully translated to Chinese and culturally adapted for Chinese populations. This pilot study demonstrates satisfactory preliminary comprehensibility and feasibility of the adapted instruments. However, these findings are preliminary due to the relatively small sample size and require confirmation in larger studies. A comprehensive psychometric evaluation is needed, including assessment of test-retest reliability for the full STAB, construct and criterion validity, diagnostic accuracy, and responsiveness to change. Definitive conclusions regarding their clinical readiness can only be drawn upon the completion of such validation.

## Supplementary Material

Supplementary material associated with this article can be found, in the online version, at https://files.jofph.com/ files/article/2031964825389547520/attachment/ Supplementary%20material.zip.




