# Supplementary material for: Translation, cultural adaptation, and pilot testing of the standardized tool for the assessment of bruxism and the bruxism screener in China
Source: J Oral Facial Pain Headache. 2026 Mar 12;40(2):54–63. doi: 10.22514/jofph.2026.020 (PMC13036617; doi:10.22514/jofph.2026.020)
Supplement: Supplementary file 1 [file Supplementary-material-1.docx]

Supplementary material 1

Supplementary Table 1.1. BruxScreen detailed results: Jaw symptoms.

| Item | Symptom | Never | Sometimes | Regularly | Often | Always | Don’t know |
| --- | --- | --- | --- | --- | --- | --- | --- |
| Upon awakening | | | | | | | |
|  | Pain | 19 (95%) | 1 (5%) | 0 | 0 | 0 | 0 |
|  | Unpleasant | 18 (90%) | 2 (10%) | 0 | 0 | 0 | 0 |
|  | Sensitivity | 19 (95%) | 1 (5%) | 0 | 0 | 0 | 0 |
|  | Tiredness | 14 (70%) | 5 (25%) | 0 | 1 (5%) | 0 | 0 |
|  | Tension | 17 (85%) | 3 (15%) | 0 | 0 | 0 | 0 |
|  | Stiffness | 17 (85%) | 3 (15%) | 0 | 0 | 0 | 0 |
| Any other times | | | | | | | |
|  | Pain | 19 (95%) | 1 (5%) | 0 | 0 | 0 | 0 |
|  | Unpleasant | 19 (95%) | 1 (5%) | 0 | 0 | 0 | 0 |
|  | Sensitivity | 19 (95%) | 1 (5%) | 0 | 0 | 0 | 0 |
|  | Tiredness | 14 (70%) | 6 (30%) | 0 | 0 | 0 | 0 |
|  | Tension | 17 (85%) | 3 (15%) | 0 | 0 | 0 | 0 |
|  | Stiffness | 17 (85%) | 3 (15%) | 0 | 0 | 0 | 0 |
| Mouth opening or chewing during meals | | | | | | | |
|  | Pain | 17 (85%) | 3 (15%) | 0 | 0 | 0 | 0 |
|  | Unpleasant | 19 (95%) | 1 (5%) | 0 | 0 | 0 | 0 |
|  | Sensitivity | 17 (85%) | 3 (15%) | 0 | 0 | 0 | 0 |
|  | Tiredness | 10 (50%) | 10 (50%) | 0 | 0 | 0 | 0 |
|  | Tension | 15 (75%) | 5 (25%) | 0 | 0 | 0 | 0 |
|  | Stiffness | 16 (80%) | 4 (20%) | 0 | 0 | 0 | 0 |
| Mouth opening or chewing at other times | | | | | | | |
|  | Pain | 17 (85%) | 3 (15%) | 0 | 0 | 0 | 0 |
|  | Unpleasant | 20 (100%) | 0 | 0 | 0 | 0 | 0 |
|  | Sensitivity | 17 (85%) | 3 (15%) | 0 | 0 | 0 | 0 |
|  | Tiredness | 15 (75%) | 5 (25%) | 0 | 0 | 0 | 0 |
|  | Tension | 15 (75%) | 5 (25%) | 0 | 0 | 0 | 0 |
|  | Stiffness | 15 (75%) | 5 (25%) | 0 | 0 | 0 | 0 |

Supplementary Table 1.2. BruxScreen detailed results: Inspections.

| Item | Absent | Present |
| --- | --- | --- |
| Masseter hypertrophy (relaxed) | 18 (90%) | 2 (10%) |
| Masseter hypertrophy (contracted) | 17 (85%) | 3 (15%) |
| Lip indentations | 20 (100%) | 0 |
| Cheek linea alba | 3 (15%) | 17 (85%) |
| Tongue indentations | 7 (35%) | 13 (65%) |
| Tongue traumatic lesions | 20 (100%) | 0 |
| Alveolar bone exostoses | 20 (100%) | 0 |

Supplementary Table 1.3. BruxScreen detailed results: Tooth wear.

| Sextant | Mean |
| --- | --- |
| Sextant 1-occlusal | 0.2 |
| Sextant 2-incisal | 0.4 |
| Sextant 2-palatal | 0 |
| Sextant 3-occlusal | 0.2 |
| Sextant 4-occlusal | 0.2 |
| Sextant 5-incisal | 0.8 |
| Sextant 6-occlusal | 0.2 |

Supplementary Table 1.4. BruxScreen detailed results: Tooth wear etiology.

| Tooth Wear Etiology | Mainly mechanical | Mainly chemical | Both |
| --- | --- | --- | --- |
| Count | 20 (100%) | 0 | 0 |
