# Supplementary material for: Translation, cultural adaptation, and pilot testing of the standardized tool for the assessment of bruxism and the bruxism screener in China
Source: J Oral Facial Pain Headache. 2026 Mar 12;40(2):54–63. doi: 10.22514/jofph.2026.020 (PMC13036617; doi:10.22514/jofph.2026.020)
Supplement: Supplementary file 2 [file Supplementary-material-2.pdf]

2.1 the Bruxism Screener (Bruxcreen) original document

APPENDIX 1

BRUXSCREEN-Q

Bruxism Screener (BruxScreen)

Part I: self-report questionnaire

Explanation

This questionnaire assesses the possible presence of bruxism and its related jaw symptoms. Bruxism is an activity of the jaw muscles that expresses itself in two different ways: as light or firm clenching of the jaws and teeth, or as grinding of the teeth. Clenching is a motionless activity, while during grinding the lower jaw moves along the upper jaw. Grinding is usually accompanied by grinding sounds. Both activities can occur during sleep and while awake. Usually, bruxism is a harmless activity, but some individuals suffer from some possible consequences, like pain in the jaws, jaw-muscle stiffness, or a limited mouth opening. You are kindly requested to answer the brief questionnaire below, so that you dentist will have more information available about the possible presence of this jaw-muscle activity. Please hand the fully completed questionnaire to your dentist, who will subsequently perform a brief inspection of your jaws and teeth as to confirm the presence of the conditions that you have indicated on this questionnaire.

Thank you for your help!
Your dentist

1. Bruxism

a. How often do you clench your teeth during sleep?
☐ never, ☐ sometimes, ☐ regularly, ☐ often, ☐ always, ☐ don't know

b. How often do you grind your teeth during sleep?
☐ never, ☐ sometimes, ☐ regularly, ☐ often, ☐ always, ☐ don't know

c. How often do you clench your teeth while awake?
☐ never, ☐ sometimes, ☐ regularly, ☐ often, ☐ always, ☐ don't know

d. How often do you grind your teeth while awake?
☐ never, ☐ sometimes, ☐ regularly, ☐ often, ☐ always, ☐ don't know

e. How often do you lightly press, touch, or hold teeth together while awake other than while eating (that is, contact between upper and lower teeth)?
☐ never, ☐ sometimes, ☐ regularly, ☐ often, ☐ always, ☐ don't know

f. How often do you firmly hold, tighten, or tense muscles while awake without clenching or bringing teeth together?
☐ never, ☐ sometimes, ☐ regularly, ☐ often, ☐ always, ☐ don't know

2. Jaw symptoms

a. How often do you experience pain/unpleasantness/sensitivity/tiredness/tension/stiffness in your temple, face, jaw, or jaw joint?

	Pain	Unpleasantness	Sensitivity	Tiredness	Tension	Stiffness
Upon awakening	☐ never<br>☐ sometimes<br>☐ regularly<br>☐ often<br>☐ always<br>☐ don't know	☐ never<br>☐ sometimes<br>☐ regularly<br>☐ often<br>☐ always<br>☐ don't know	☐ never<br>☐ sometimes<br>☐ regularly<br>☐ often<br>☐ always<br>☐ don't know	☐ never<br>☐ sometimes<br>☐ regularly<br>☐ often<br>☐ always<br>☐ don't know	☐ never<br>☐ sometimes<br>☐ regularly<br>☐ often<br>☐ always<br>☐ don't know	☐ never<br>☐ sometimes<br>☐ regularly<br>☐ often<br>☐ always<br>☐ don't know
Any other time	☐ never<br>☐ sometimes<br>☐ regularly<br>☐ often<br>☐ always<br>☐ don't know	☐ never<br>☐ sometimes<br>☐ regularly<br>☐ often<br>☐ always<br>☐ don't know	☐ never<br>☐ sometimes<br>☐ regularly<br>☐ often<br>☐ always<br>☐ don't know	☐ never<br>☐ sometimes<br>☐ regularly<br>☐ often<br>☐ always<br>☐ don't know	☐ never<br>☐ sometimes<br>☐ regularly<br>☐ often<br>☐ always<br>☐ don't know	☐ never<br>☐ sometimes<br>☐ regularly<br>☐ often<br>☐ always<br>☐ don't know

13652842, 2023, 0, Downloaded from https://onlinelibrary.wiley.com/doi/10.1111/joor.13442 by Wiley Online Library, Wiley Online Library on [10/12/2023]. See the Terms and Conditions (https://onlinelibrary.wiley.com/terms-and-conditions) on Wiley Online Library for rules of use; OA articles are governed by the applicable Creative Commons License

b. How often do you experience pain/unpleasantness/sensitivity/tiredness/tension/stiffness in your temple, face, jaw, or jaw joint when you open your mouth or chew?

	Pain	Unpleasantness	Sensitivity	Tiredness	Tension	Stiffness
During meals	☐ never<br>☐ sometimes<br>☐ regularly<br>☐ often<br>☐ always<br>☐ don't know	☐ never<br>☐ sometimes<br>☐ regularly<br>☐ often<br>☐ always<br>☐ don't know	☐ never<br>☐ sometimes<br>☐ regularly<br>☐ often<br>☐ always<br>☐ don't know	☐ never<br>☐ sometimes<br>☐ regularly<br>☐ often<br>☐ always<br>☐ don't know	☐ never<br>☐ sometimes<br>☐ regularly<br>☐ often<br>☐ always<br>☐ don't know	☐ never<br>☐ sometimes<br>☐ regularly<br>☐ often<br>☐ always<br>☐ don't know
Any other time	☐ never<br>☐ sometimes<br>☐ regularly<br>☐ often<br>☐ always<br>☐ don't know	☐ never<br>☐ sometimes<br>☐ regularly<br>☐ often<br>☐ always<br>☐ don't know	☐ never<br>☐ sometimes<br>☐ regularly<br>☐ often<br>☐ always<br>☐ don't know	☐ never<br>☐ sometimes<br>☐ regularly<br>☐ often<br>☐ always<br>☐ don't know	☐ never<br>☐ sometimes<br>☐ regularly<br>☐ often<br>☐ always<br>☐ don't know	☐ never<br>☐ sometimes<br>☐ regularly<br>☐ often<br>☐ always<br>☐ don't know

c. How often does your jaw lock or become stuck?

(i) During meals:
☐ never, ☐ sometimes, ☐ regularly, ☐ often, ☐ always, ☐ don't know

(ii) Any other time:
☐ never, ☐ sometimes, ☐ regularly, ☐ often, ☐ always, ☐ don't know

13652842, 2023, 0, Downloaded from https://onlinelibrary.wiley.com/doi/10.1111/joor.13442. By Wiley Online Library, Wiley Online Library on [10/12/2023]. See the Terms and Conditions (https://onlinelibrary.wiley.com/terms-and-conditions) on Wiley Online Library for rules of use; OA articles are governed by the applicable Creative Commons License

BRUXSCREEN-C

Bruxism Screener (BruxScreen)

Part II: clinical assessment form

Explanation

This clinical assessment tool aims at identifying signs that may be associated with bruxism. First, the facial outline is inspected for possible hypertrophy of the masseter muscles, both while the muscles are relaxed and when they are contracted. In case of doubt, select 'absent'. Second, the oral cavity is inspected for signs that may be associated with bruxism. Also in this case, when in doubt, select 'absent'. Third, the teeth are inspected for tooth wear. Please follow the scoring rules as indicated. In case of doubt between two scores, select the lower one. In addition, please indicate if the observed tooth wear is mainly mechanical (i.e. due to tooth-to-tooth contact), mainly chemical, or both.

1. Extra-oral inspection

a. Masseter muscle hypertrophy (observed while the muscles are relaxed)
☐ absent, ☐ present

b. Masseter muscle hypertrophy (observed while the muscles are contracted)
☐ absent, ☐ present

2. Intra-oral inspection of non-dental tissues

a. Lip (indentations) b. Cheek (linea alba) c. Tongue (indentations)
☐ absent, ☐ present ☐ absent, ☐ present ☐ absent, ☐ present

b. Tongue (traumatic lesions) e. Alveolar bone (exostoses/tori)
☐ absent, ☐ present ☐ absent, ☐ present

3. Intra-oral inspection of dental tissues

a. Occlusal/incisal wear per sextant
(0) no visible wear, (1) visible wear within the enamel, (2) visible wear with dentin exposure and loss of clinical crown height of ≤1/3, (3) loss of crown height >1/3 but <2/3, (4) loss of crown height ≥2/3

b. Palatal wear in sextant no. 2
(0) no visible wear, (1) wear confined to the enamel, (2) wear into the dentine

Sextant 1 – occlusal	Sextant 2 – incisal	Sextant 3 – occlusal
0 1 2 3 4	0 1 2 3 4	0 1 2 3 4
	Sextant 2 - palatal	
	0 1 2	
Sextant 6 – occlusal	Sextant 5 – incisal	Sextant 4 – occlusal
0 1 2 3 4	0 1 2 3 4	0 1 2 3 4

c. The observed tooth wear is:
☐ mainly mechanical, ☐ mainly chemical, ☐ both mechanical and chemical

13652842, 2023, 0, Downloaded from https://onlinelibrary.wiley.com/doi/10.1111/joor.13442. By Wiley Online Library, Wiley Online Library on [10/12/2023]. See the Terms and Conditions (https://onlinelibrary.wiley.com/terms-and-conditions) on Wiley Online Library for rules of use; OA articles are governed by the applicable Creative Commons License

2.2 the Standardized Tool for the Assessment of Bruxism (STAB) original document

Standardized Tool for the Assessment of Bruxism

STAB

Full Instrument

ENGLISH VERSION

August 2022

13652842, 2023, 0, Downloaded from https://onlinelibrary-wiley-com-443.webvpn.bjmu.edu.cn/doi/10.1111/joor.13411. By Peking University Health, Wiley Online Library on [25/11/2023]. See the Terms and Conditions (https://onlinelibrary-wiley-com-443.webvpn.bjmu.edu.cn/terms-and-conditions) on Wiley Online Library for rules of use; OA articles are governed by the applicable Creative Commons License

Index of contents

Demographics Questionnaire Page 3

Axis A – Assessment of Bruxism Status and Consequences 4

Subject Based Assessment (SBA) – Self Report 5

A1. Sleep Bruxism Report 5

A2. Awake Bruxism Report 6

A3. Patient's Complaints 8

Clinically Based Assessment (CBA) – Examiner's Report 11

A4. Joints and Muscles 11

A5. Intraoral and Extraoral Tissues 12

A6. Teeth and Restorations 13

Instrumentally Based Assessment (IBA) – Technology Report 15

A7. Sleep Bruxism 15

A8. Awake Bruxism 16

A9. Additional Instruments 17

Axis B – Risk and Etiological Factors and Comorbid Conditions 18

B1. Psychosocial Assessment 19

B2. Concurrent Sleep-related Conditions Assessment 21

B3. Concurrent Non-Sleep Conditions Assessment 22

B4. Prescribed Medications and Use of Substances Assessment 26

B5. Additional Factors Assessment 28

13652842, 2023, 0, Downloaded from https://onlinelibrary-wiley-com-443.webvpn.bjmu.edu.cn/doi/10.1111/joor.13411. By Peking University Health, Wiley Online Library on [25/11/2023]. See the Terms and Conditions (https://onlinelibrary-wiley-com-443.webvpn.bjmu.edu.cn/terms-and-conditions) on Wiley Online Library for rules of use; OA articles are governed by the applicable Creative Commons License

Standardized Tool for the Assessment of Bruxism (STAB)

Demographics Questionnaire

Sex
Male
Female
Unspecified/Other

**Age**	**Height**	**Weight**
___________	___________	___________

What is your current marital status?
Married
Living as married
Divorced
Separated
Widowed
Never married

What is the highest grade or level of schooling that you have completed?
Compulsory school (<16 years)
Secondary school (e.g., high school) up to 18 years
Some University (no degree)
University graduate
Post-graduate level

13652842, 2023, 0, Downloaded from https://onlinelibrary-wiley-com-443.webvpn.bjmu.edu.cn/doi/10.1111/joor.13411. By Peking University Health, Wiley Online Library on [25/11/2023]. See the Terms and Conditions (https://onlinelibrary-wiley-com-443.webvpn.bjmu.edu.cn/terms-and-conditions) on Wiley Online Library for rules of use; OA articles are governed by the applicable Creative Commons License

AXIS A - Assessment of Bruxism Status and Consequences

13652842, 2023, 0, Downloaded from https://onlinelibrary-wiley-com-443.webvpn.bjmu.edu.cn/doi/10.1111/joor.13411. By Peking University Health, Wiley Online Library on [25/11/2023]. See the Terms and Conditions (https://onlinelibrary-wiley-com-443.webvpn.bjmu.edu.cn/terms-and-conditions) on Wiley Online Library for rules of use; OA articles are governed by the applicable Creative Commons License

Subject Based Assessment (SBA)
Self Report

A1. Sleep Bruxism report

A1.1 SLEEP BRUXISM QUESTION
How often do you clench or grind your teeth when asleep based on the last month (based on any information you may have)?
None of the time
Less than one night/ month
1-3 nights / month
1-3 nights/ week
4-7 nights / week
Don't know

A1.1.1 SLEEP BRUXISM HISTORY QUESTION
Did you use to clench or grind your teeth when asleep in the past, based on any information you have?
No
Yes
Don't know

13652842, 2023, 0, Downloaded from https://onlinelibrary-wiley-com-443.webvpn.bjmu.edu.cn/doi/10.1111/joor.13411. By Peking University Health, Wiley Online Library on [25/11/2023]. See the Terms and Conditions (https://onlinelibrary-wiley-com-443.webvpn.bjmu.edu.cn/terms-and-conditions) on Wiley Online Library for rules of use; OA articles are governed by the applicable Creative Commons License

A2. Awake Bruxism report

A2.1 AWAKE TEETH GRINDING QUESTION
How often do you grind your teeth together during waking hours, based on the last month?
None of the time
A little of the time
Some of the time
Most of the time
All of the time
Don’t know

A2.1.1 AWAKE TEETH GRINDING HISTORY QUESTION
Did you use to grind your teeth together during waking hours in the past?

No
Yes
Don’t know

A2.2 AWAKE TEETH CLENCHING QUESTION
How often do you clench your teeth together during waking hours, based on the last month?

None of the time
A little of the time
Some of the time
Most of the time
All of the time
Don’t know

A2.2.1 AWAKE TEETH CLENCHING HISTORY QUESTION
Did you use to clench your teeth together during waking hours in the past?

No
Yes
Don’t know

A2.3 AWAKE TEETH CONTACT QUESTION
How often do you press, touch, or hold your teeth together other than while eating (that is, contact between upper and lower teeth), based on the last month?
None of the time
A little of the time
Some of the time
Most of the time
All of the time
Don’t know

13652842, 2023, 0, Downloaded from https://onlinelibrary-wiley-com-443.webvpn.bjmu.edu.cn/doi/10.1111/joor.13411. By Peking University Health, Wiley Online Library on [25/11/2023]. See the Terms and Conditions (https://onlinelibrary-wiley-com-443.webvpn.bjmu.edu.cn/terms-and-conditions) on Wiley Online Library for rules of use; OA articles are governed by the applicable Creative Commons License

A2.3.1 AWAKE TEETH CONTACT HISTORY QUESTION

Did you use to press, touch, or hold your teeth together other than while eating (that is, contact between upper and lower teeth) in the past?

No
Yes
Don’t know

A2.4 AWAKE MANDIBLE BRACING QUESTION

How often do you hold, tighten, or tense your muscles without clenching or bringing teeth together, based on the last month?

None of the time
A little of the time
Some of the time
Most of the time
All of the time
Don’t know

A2.4.1 AWAKE MANDIBLE BRACING HISTORY QUESTION

Did you use to hold, tighten, or tense your muscles without clenching or bringing teeth together in the past?

No
Yes
Don’t know

13652842, 2023, 0, Downloaded from https://onlinelibrary-wiley-com-443.webvpn.bjmu.edu.cn/doi/10.1111/joor.13411. By Peking University Health, Wiley Online Library on [25/11/2023]. See the Terms and Conditions (https://onlinelibrary-wiley-com-443.webvpn.bjmu.edu.cn/terms-and-conditions) on Wiley Online Library for rules of use; OA articles are governed by the applicable Creative Commons License

A3. Patient's complaints

TEMPOROMANDIBULAR DISORDERS

A3.1 TMD PAIN
In the last 30 days, how long did any pain last in your jaw or temple area on either side?
No pain
Pain comes and goes
Pain is always present

A3.2 PAIN OR STIFFNESS ON AWAKENING
In the last 30 days, have you had pain or stiffness in your jaw on awakening?
No
Yes

A3.3 CLOSED LOCK
In the last 30 days, have you had your jaw locked or caught, even for a moment, so it would not open ALL THE WAY?
No
Yes

A3.4 PAIN CHANGE WITH ACTIVITIES
In the last 30 days, did the following activities change any pain (that is, make it better or make it worse) in your jaw or temple on either side?
Chewing hard or tough food
Opening your mouth or moving your jaw forward or to the side
Jaw habits (e.g., holding teeth together, clenching, grinding, chewing gum)
Other jaw activities such as talking, kissing, or yawning

A3.5 JAW JOINT NOISES
In the last 30 days, have you had any jaw joint noise(s) when you moved or used your jaw?
No
Yes

A3.6 WAKETIME MUSCLE PAIN
In the last 30 days, did you have jaw muscle pain during any of the following times of the day?
Between waking up and breakfast
Between breakfast and lunch
Between lunch and dinner
Between dinner and bedtime

13652842, 2023, 0, Downloaded from https://onlinelibrary-wiley-com-443.webvpn.bjmu.edu.cn/doi/10.1111/joor.13411 by Peking University Health, Wiley Online Library on [25/ /2023]. See the Terms and Conditions (https://onlinelibrary-wiley-com-443.webvpn.bjmu.edu.cn/terms-and-conditions) on Wiley Online Library for rules of use; OA articles are governed by the applicable Creative Commons License

A3.6.1 WAKETIME MUSCLE TIREDNESS OR FATIGUE

In the last 30 days, did you have jaw muscle stiffness or sensation or tiredness or fatigue during any of the following times of the day?

Between waking up and breakfast
Between breakfast and lunch
Between lunch and dinner
Between dinner and bedtime

A3.7 AWAKENING SYMPTOMS QUESTION

Are you aware of any of the following symptoms upon awakening?

Sensation of fatigue, soreness or tightness of your jaw
Feeling that your teeth are clenched or that your mouth is sore
Aching of your temples
Feeling of tension in your jaw joint upon awakening and feeling that you have to move your lower jaw to release it
Difficulty in opening mouth wide upon awakening
Hearing or feeling a click in your jaw joint upon awakening that disappears afterwards

HEADACHE

A3.8 HEADACHE

In the past 30 days, have you had any headache that included the temple areas of your head?

No
Yes

If yes – how many days?

TOOTH WEAR

A3.9 TOOTH WEAR

Do you experience any of the following symptoms because of the existing tooth wear?

Sensitivity and/or pain
Functional problems (difficulties chewing and eating)
Deterioration of esthetic appearance (compromised dental attractiveness)
Crumbling of dental hard tissue and restorations
Phonetic impairment

TINNITUS

A3.10 TINNITUS

Do you have noises or ringing in your ears (tinnitus)?

No
Yes

13652842, 2023, 0, Downloaded from https://onlinelibrary-wiley-com-443.webvpn.bjmu.edu.cn/doi/10.1111/joor.13411. By Peking University Health, Wiley Online Library on [25/11/2023]. See the Terms and Conditions (https://onlinelibrary-wiley-com-443.webvpn.bjmu.edu.cn/terms-and-conditions) on Wiley Online Library for rules of use; OA articles are governed by the applicable Creative Commons License

XEROSTOMIA AND DROOLING

A3.11 XEROSTOMIA

Does your mouth feel dry?

Never
Occasionally
Often

A3.12 DROOLING

Do you experience loss of saliva during the night?

I do not experience loss of saliva during the night at all
My pillow sometimes gets wet during the night
My pillow regularly gets wet during the night
My pillow always gets wet during the night
Every night my pillow and other bedclothes get wet

13652842, 2023, 0, Downloaded from https://onlinelibrary-wiley-com-443.webvpn.bjmu.edu.cn/doi/10.1111/joor.13411. By Peking University Health, Wiley Online Library on [25/11/2023]. See the Terms and Conditions (https://onlinelibrary-wiley-com-443.webvpn.bjmu.edu.cn/terms-and-conditions) on Wiley Online Library for rules of use; OA articles are governed by the applicable Creative Commons License

Clinically Based Assessment (CBA)
Examiner Report (FOR EXAMINER'S USE)

A4. Joints and muscles

A4.1 TMD DIAGNOSES (Optional Item)
Mark the presence of the following diagnoses

A4.1.1. PAIN DISORDERS*
*Please fill this section only if the item A3.3 (TMD Pain Screener) is positively endorsed by the patient
Myalgia
Myofascial pain with spreading
Myofascial pain with referral
Right arthralgia
Left arthralgia
Headache attributed to TMD

A4.1.2. RIGHT TMJ DISORDERS*
*Please specify if diagnosis is based upon clinical or imaging assessment
Disc displacement with reduction
Disc displacement with reduction, with intermittent locking
Disc displacement without reduction with limited opening
Disc displacement without reduction without limited opening
Degenerative joint disease
Subluxation

A4.1.3. LEFT TMJ DISORDERS*
Please specify if diagnosis is based upon clinical or imaging assessment
Disc displacement with reduction
Disc displacement with reduction, with intermittent locking
Disc displacement without reduction with limited opening
Disc displacement without reduction without limited opening
Degenerative joint disease
Subluxation

A4.2 MASSETER MUSCLE HYPERTROPHY
Mark the presence of masseter hypertrophy:
Left
Right

13652842, 2023, 0, Downloaded from https://onlinelibrary-wiley-com-443.webvpn.bjmu.edu.cn/doi/10.1111/joor.13411. By Peking University Health, Wiley Online Library on [25/11/2023]. See the Terms and Conditions (https://onlinelibrary-wiley-com-443.webvpn.bjmu.edu.cn/terms-and-conditions) on Wiley Online Library for rules of use; OA articles are governed by the applicable Creative Commons License

A5. Intra- and Extra-oral tissues

A5.1 SOFT AND BONE TISSUES

Mark the presence of the following signs:

Linea alba	Left	Right	
Lip impression	Upper	Lower	
Tongue scalloping	Right	Front	Left
Tongue ulceration*	Right	Front	Left
Alveolar bone exostosis	Mandible (Buccal/Lingual)	Maxilla (Buccal/Lingual/Midpalatal)	

*If positive for firm, indurated, rolled borders – red flag for further urgent investigation

A5.2 MODIFIED FRIEDMAN TONGUE POSITION

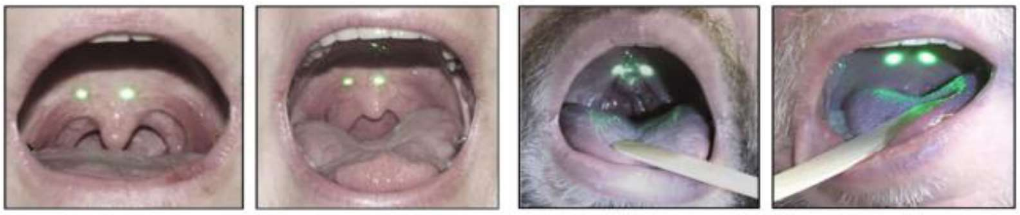

Category I: No phonation/ tongue depressor – uvula and palatal arch visible

Category II: With phonation – uvula and palatal arch visible

Category III: With tongue depressor – uvula and palatal arch visible

Category IV: With phonation/ tongue depressor – uvula and palatal arch not visible

A5.3 SKELETAL CLASS (Optional Item)

Class 1
Class 2
Class 3

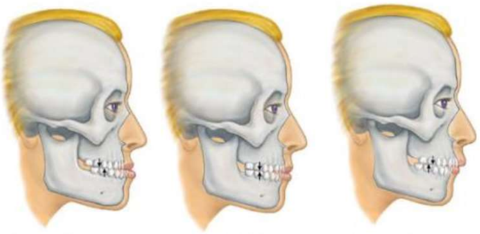

Skeletal Class III Facial Profile | Skeletal Class I Facial Profile | Skeletal Class II Facial Profile

A5.4 SKELETAL PROFILE (Optional Item)

Normodivergent profile (medlium gonial angle)
Hypodivergent profile (low gonial angle)
Hyperdivergent profile (high gonial angle)

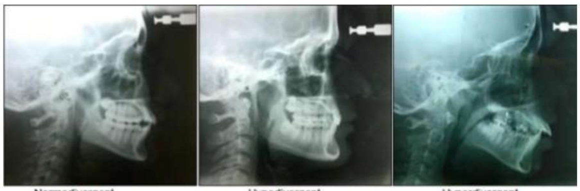

Normodivergent | Hypodivergent | Hyperdivergent

13652842, 2023, 0, Downloaded from https://onlinelibrary-wiley-com-443.webvpn.bjmu.edu.cn/doi/10.1111/joor.13411. By Peking University Health, Wiley Online Library on [25/11/2023]. See the Terms and Conditions (https://onlinelibrary-wiley-com-443.webvpn.bjmu.edu.cn/terms-and-conditions) on Wiley Online Library for rules of use; OA articles are governed by the applicable Creative Commons License

A6. Teeth and restorations

A6.1 TOOTH WEAR SCREENING – QUANTIFICATION
Indicate the highest tooth wear score per sextant

	Sextant 1	Sextant 2	Sextant 3	Sextant 4	Sextant 5	Sextant 6
Occlusal/Incisal						
Palatal						

A.6.1.1 TOOTH WEAR QUALIFICATION*
*When during quantification a grade ≥ 2 is detected, qualification is needed.
Clinical signs indicating the influence of mechanical factors:

Shiny facets, flat and glossy
Enamel and dentin wear at the same rate
Matching wear on occluding surfaces, corresponding features at the antagonistic teeth
Fracture of cusps or restorations
Impressions in cheek, tongue and/or lip
Located at cervical areas of the teeth, Non Carious Cervical Lesions (NCCL)
Buccal/cervical lesions more wide than deep, Non Carious Cervical Lesions (NCCL)
Cervical areas of premolars and cuspids are affected
Cracks within the enamel
Torus mandibulae

A6.2 PERIODONTAL AND DENTAL EXAMINATION
Indicate the number of teeth with the following signs

	Sextant 1	Sextant 2	Sextant 3	Sextant 4	Sextant 5	Sextant 6
Mobility						
Thermal sensitivity						
Discomfort/pain on biting						
Fractured teeth						

13652842, 2023, 0, Downloaded from https://onlinelibrary-wiley-com-443.webvpn.bjmu.edu.cn/doi/10.1111/joor.13411. By Peking University Health, Wiley Online Library on [25/11/2023]. See the Terms and Conditions (https://onlinelibrary-wiley-com-443.webvpn.bjmu.edu.cn/terms-and-conditions) on Wiley Online Library for rules of use; OA articles are governed by the applicable Creative Commons License

A6.3 RESTORATIONS

Indicate the number of teeth/implants with the following signs

	Sextant 1	Sextant 2	Sextant 3	Sextant 4	Sextant 5	Sextant 6
Lost/broken fillings						
Scratched restorations						
Ceramic fractures						
Mobile implants						
Implant fractures						
Implant screw loosening						

A6.4 ORAL APPLIANCE EVALUATION (If hard resin splint is used by the patient)

Mark the presence of the following signs:

Prevalence of grinding marks (i.e., stripes)	Right	Front	Left
Prevalence of clenching marks (i.e., circle-like spots)	Right	Front	Left
Combination of grinding and clenching marks	Right	Front	Left
Fractures or perforations	Right	Front	Left

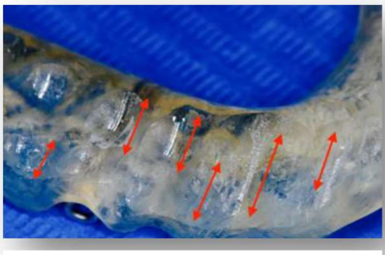

Example of grinding marks

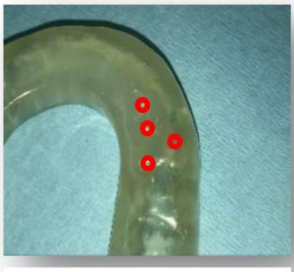

Example of clenching marks.

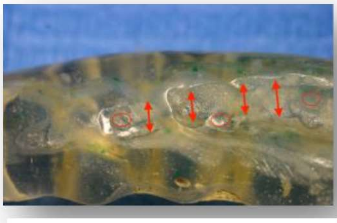

Example of combined marks.

13652842, 2023, 0, Downloaded from https://onlinelibrary-wiley-com-443.webvpn.bjmu.edu.cn/doi/10.1111/joor.13411. By Peking University Health, Wiley Online Library on [25/11/2023]. See the Terms and Conditions (https://onlinelibrary-wiley-com-443.webvpn.bjmu.edu.cn/terms-and-conditions) on Wiley Online Library for rules of use; OA articles are governed by the applicable Creative Commons License

Instrumentally Based Assessment (IBA) Technology Report

A7. Sleep Bruxism

A7.1 ELECTROMYOGRAPHY

Number of masseter events over 10% MVC ________________
Bruxism time index (if available) ________________
Bruxism work index (if available) ________________

A7.2 POLYSOMNOGRAPHY (Optional)

Number of arousal-related SB events ________________
Number of arousal-unrelated SB events ________________
Bruxism time index (if available) ________________
Bruxism work index (if available) ________________

A7.3 OTHER METHODS (Optional)

Smartphone application scores for grinding sounds

A7.4 APPLIANCE WITH SENSORS (Optional)

Number of events of bite pressure

13652842, 2023, 0, Downloaded from https://onlinelibrary-wiley-com-443.webvpn.bjmu.edu.cn/doi/10.1111/joor.13411. By Peking University Health, Wiley Online Library on [25/11/2023]. See the Terms and Conditions (https://onlinelibrary-wiley-com-443.webvpn.bjmu.edu.cn/terms-and-conditions) on Wiley Online Library for rules of use; OA articles are governed by the applicable Creative Commons License

A8. Awake Bruxism

A8.1 ECOLOGICAL MOMENTARY ASSESSMENT - One week report

CONDITION	Week 1 (in %)	Additional week(s) (in %)
Relaxed jaw muscles (no teeth contact)		
Mandible bracing (no teeth contact)		
Teeth Contact (light steady touching)		
Teeth Clenching (strong steady touching)		
Teeth Grinding		

A8.2 WAKE-TIME ELECTROMYOGRAPHY

Number of masseter events over 10% MVC ______________
Bruxism time index ______________
Bruxism work index ______________

A8.3 OTHER METHODS: To be determined

A9. Additional instruments

A9.1. INTRAORAL ACIDITY (Optional)

REST SALIVARY PH ____________________
REST SALVIARY FLOW ____________________
STIMULATED SALIVARY PH (paraffin) ____________________
STIMULATED SALIVARY FLOW ____________________

13652842, 2023, 0, Downloaded from https://onlinelibrary-wiley-com-443.webvpn.bjmu.edu.cn/doi/10.1111/joor.13411. By Peking University Health, Wiley Online Library on [25/11/2023]. See the Terms and Conditions (https://onlinelibrary-wiley-com-443.webvpn.bjmu.edu.cn/terms-and-conditions) on Wiley Online Library for rules of use; OA articles are governed by the applicable Creative Commons License

AXIS B – Risk and Etiological factors and comorbid conditions

13652842, 2023, 0, Downloaded from https://onlinelibrary-wiley-com-443.webvpn.bjmu.edu.cn/doi/10.1111/joor.13411. By Peking University Health, Wiley Online Library on [25/11/2023]. See the Terms and Conditions (https://onlinelibrary-wiley-com-443.webvpn.bjmu.edu.cn/terms-and-conditions) on Wiley Online Library for rules of use; OA articles are governed by the applicable Creative Commons License

B1. Psychosocial assessment
Self Report

B1.1 ANXIETY and DEPRESSION SCREENING

In the last two weeks, how often have you been bothered by the following problems?

1. Feeling nervous, anxious or on edge
Not at all
Several days
More than half the days
Nearly every day

2. Not being able to stop or control worrying
Not at all
Several days
More than half the days
Nearly every day

3. Little interest or pleasure in doing things
Not at all
Several days
More than half the days
Nearly every day

4. Feeling down, depressed, or hopeless
Not at all
Several days
More than half the days
Nearly every day

13652842, 2023, 0, Downloaded from https://onlinelibrary-wiley-com-443.webvpn.bjmu.edu.cn/doi/10.1111/joor.13411. By Peking University Health, Wiley Online Library on [25/11/2023]. See the Terms and Conditions (https://onlinelibrary-wiley-com-443.webvpn.bjmu.edu.cn/terms-and-conditions) on Wiley Online Library for rules of use; OA articles are governed by the applicable Creative Commons License

B1.2 COPING

Consider how well the following statements describe your behaviors and actions

1. I look for creative ways to alter difficult situations
Does not describe me at all
Does not describe me
Neutral
Describes me
Describes me very well

2. Regardless of what happens to me, I believe can control my reaction to it
Does not describe me at all
Does not describe me
Neutral
Describes me
Describes me very well

3. I believe I can grow in positive ways by dealing with difficult situations
Does not describe me at all
Does not describe me
Neutral
Describes me
Describes me very well

4. I actively ook for ways to replace the losses I encounter in life
Does not describe me at all
Does not describe me
Neutral
Describes me
Describes me very well

13652842, 2023, 0, Downloaded from https://onlinelibrary-wiley-com-443.webvpn.bjmu.edu.cn/doi/10.1111/joor.13411 by Peking University Health, Wiley Online Library on [25/11/2023]. See the Terms and Conditions (https://onlinelibrary-wiley-com-443.webvpn.bjmu.edu.cn/terms-and-conditions) on Wiley Online Library for rules of use; OA articles are governed by the applicable Creative Commons License

B2. Concurrent sleep-related conditions assessment
Self Report

B2.1 SLEEP APNEA SCREENING

Please mark which of the following questions is answered positively

Do you snore loudly?
Do you often feel tired, fatigued or sleepy during daytime?
Has anyone observed you stop breathing or choking/gasping during sleep?
Do you have or are being treated for high blood pressure?
Body mass Index more than 35?
Age older than 50?
Neck size large (43 cm or larger for males; 41 cm or larger for females)?
Gender=male?

B2.2 INSOMNIA SCREENING

Indicate which of the following statements can be applied to you

I have difficulty falling asleep
Thoughts race through my mind and prevent me from sleeping
I anticipate a problem with sleep several times a week
I wake up and cannot go back to sleep
I worry about things and have problems relaxing
I wake up earlier in the morning than I would like to
I lie awake for half an hour or more before I fall asleep

B2.3 PERIODIC LIMB MOVEMENT DISORDER and RESTLESS LEG SYNDROME SCREENING

Indicate which of the following statements can be applied to you

Other than when exercising, I still experience muscle tension in my legs
I have noticed (or other have commented) that parts of my body jerk during sleep
I have been told that I kick at night
When trying to go sleep, I experience an aching or crawling sensation in my legs
I experience leg pain and cramps at night
Sometimes I can’t keep my legs still at night. I just have to move them to feel comfortable
Even though I slept during the night, I feel sleepy during the day

B2.4 ORAL BEHAVIORS - SLEEP POSITION

How often do you sleep in a position that puts pressure on the jaw, based on the last month?

None of the time
A little of the time
Some of the time
Most of the time
All of the time
Don’t know

13652842, 2023, 0, Downloaded from https://onlinelibrary-wiley-com-443.webvpn.bjmu.edu.cn/doi/10.1111/joor.13411. By Peking University Health, Wiley Online Library on [25/11/2023]. See the Terms and Conditions (https://onlinelibrary-wiley-com-443.webvpn.bjmu.edu.cn/terms-and-conditions) on Wiley Online Library for rules of use; OA articles are governed by the applicable Creative Commons License

B3. Concurrent non-sleep conditions assessment
Self Report

B3.1 ORAL BEHAVIORS - ACTIVITIES DURING WAKING HOURS
How often do you do each of the following activities, based on the last month?

Q7. Hold or jut jaw forward or to the side
None of the time
A little of the time
Some of the time
Most of the time
All of the time
Don't know

Q8. Press tongue forcibly against teeth
None of the time
A little of the time
Some of the time
Most of the time
All of the time
Don't know

Q9. Place tongue between teeth
None of the time
A little of the time
Some of the time
Most of the time
All of the time
Don't know

Q10. Bite, chew or play with your tongue, cheeks or lips
None of the time
A little of the time
Some of the time
Most of the time
All of the time
Don't know

Q11. Hold jaw in rigid or tense position, such as to brace or protect the jaw
None of the time
A little of the time
Some of the time
Most of the time
All of the time
Don't know

13652842, 2023, 0, Downloaded from https://onlinelibrary-wiley-com-443.webvpn.bjmu.edu.cn/doi/10.1111/joor.13411. By Peking University Health, Wiley Online Library on [25/11/2023]. See the Terms and Conditions (https://onlinelibrary-wiley-com-443.webvpn.bjmu.edu.cn/terms-and-conditions) on Wiley Online Library for rules of use; OA articles are governed by the applicable Creative Commons License

Q12. Hold between the teeth or bite objects such as hair, pipe, pencil, pens, fingers, fingernails etc
None of the time
A little of the time
Some of the time
Most of the time
All of the time
Don’t know

Q13. Use chewing gum
None of the time
A little of the time
Some of the time
Most of the time
All of the time
Don’t know

Q14. Play musical instrument that involves use of mouth or jaw
None of the time
A little of the time
Some of the time
Most of the time
All of the time
Don’t know

Q15. Lean with your hand on the jaw, such as cupping or resting the chin in the hand
None of the time
A little of the time
Some of the time
Most of the time
All of the time
Don’t know

Q16. Chew food on one side only
None of the time
A little of the time
Some of the time
Most of the time
All of the time
Don’t know

Q17. Eating between meals
None of the time
A little of the time
Some of the time
Most of the time
All of the time
Don’t know

13652842, 2023, 0, Downloaded from https://onlinelibrary-wiley-com-443.webvpn.bjmu.edu.cn/doi/10.1111/joor.13411. By Peking University Health, Wiley Online Library on [25/11/2023]. See the Terms and Conditions (https://onlinelibrary-wiley-com-443.webvpn.bjmu.edu.cn/terms-and-conditions) on Wiley Online Library for rules of use; OA articles are governed by the applicable Creative Commons License

Q18. Sustained talking (e.g., teaching, sales, customer services)
None of the time
A little of the time
Some of the time
Most of the time
All of the time
Don't know

Q19. Singing
None of the time
A little of the time
Some of the time
Most of the time
All of the time
Don't know

Q20. Yawning
None of the time
A little of the time
Some of the time
Most of the time
All of the time
Don't know

Q21. Hold telephone between your head and shoulders
None of the time
A little of the time
Some of the time
Most of the time
All of the time
Don't know

B3.2 SMARTPHONE USE (Optional Item)
Indicate the average time/day of smartphone use

B3.3 OROFACIAL MOTOR DISORDERS
Have you been diagnosed with or do you suffer from possible signs of one of the following conditions?

Orofacial Dyskinesia
Oromandibular Dystonia
Parkinson's Disease
Huntington's Disease
Tourette's Syndrome
Hemifacial Spasms
Tardive Dyskinesia

13652842, 2023, 0, Downloaded from https://onlinelibrary-wiley-com-443.webvpn.bjmu.edu.cn/doi/10.1111/joor.13411. By Peking University Health, Wiley Online Library on [25/11/2023]. See the Terms and Conditions (https://onlinelibrary-wiley-com-443.webvpn.bjmu.edu.cn/terms-and-conditions) on Wiley Online Library for rules of use; OA articles are governed by the applicable Creative Commons License

B3.4 GASTROAESOPHAGEAL REFLUX DISEASE SCREENING
How many times per week do each of the following symptoms occur?

A. Burning feeling behind the breastbone (heartburn)
0 Days
1 Day
2-3 Days
4-7 Days

B. Stomach contents moving up to the throat or mouth (regurgitation)
0 Days
1 Day
2-3 Days
4-7 Days

C. Pain in the middle or upper stomach area
0 Days
1 Day
2-3 Days
4-7 Days

D. Nausea
0 Days
1 Day
2-3 Days
4-7 Days

E. Trouble getting a good night's sleep because of heartburn or regurgitation
0 Days
1 Day
2-3 Days
4-7 Days

F. Need for over-the-counter medicine for heartburn or regurgitation
0 Days
1 Day
2-3 Days
4-7 Days

B3.5 AUTOIMMUNE OR CONNECTIVE TISSUE DISORDERS SCREENING
Have you been diagnosed with one of the following conditions?
Rheumatoid Arthritis
Lupus
Other systemic rheumatic diseases, including fibromyalgia
Other systemic conditions, including systemic sclerosis, rheumatic polymyalgia, mixed connective disease

B3.6 ATTENTION DEFICIT HYPERACTIVITY DISORDER
Have you been diagnosed with Attention Deficit Hyperactive Disorder?
No
Yes

13652842, 2023, 0, Downloaded from https://onlinelibrary-wiley-com-443.webvpn.bjmu.edu.cn/doi/10.1111/joor.13411. By Peking University Health, Wiley Online Library on [25/11/2023]. See the Terms and Conditions (https://onlinelibrary-wiley-com-443.webvpn.bjmu.edu.cn/terms-and-conditions) on Wiley Online Library for rules of use; OA articles are governed by the applicable Creative Commons License

B4. Prescribed medications and use of substances assessment Self Report

B4.1 DRUGS
Mark if you use recreational or street drugs
If yes, please state which drugs you use for recreational purposes ____________________

B4.2 MEDICATIONS
Are you currently under one of the following medications?
Antidepressants (e.g., Selective serotonin-reuptake inhibitors)
Benzodiazepines
Neuroleptics, Antipsychotics, Antiemetics (Dopamine antagonists)
ADHD medication
Anti-allergic medication
Medical marijuana CBD
Medical marijuana TSH
Opioids
Others
If yes, please list all the medications and dosage
____

B4.3 TOBACCO
Do you smoke or use any tobacco products?
No
Yes
Quit
If yes, how many cigarettes/day do you smoke? N°_____________

B4.4 ALCOHOL
Do you ever drink alcoholic beverages (beer, wine, hard liquor)?
No
Yes
Quit
If yes, what is your approximate intake of these alcoholic beverages (glasses/day)? _______

B4.5 SOFT DRINKS
Do you regularly drink sparkling drinks (e.g., Cola – RedBull – Sprite – Fanta)?
No
Yes
Quit
If yes, what is your approximate intake (glasses/day)? __________

13652842, 2023, 0, Downloaded from https://onlinelibrary-wiley-com-443.webvpn.bjmu.edu.cn/doi/10.1111/joor.13411. By Peking University Health, Wiley Online Library on [25/11/2023]. See the Terms and Conditions (https://onlinelibrary-wiley-com-443.webvpn.bjmu.edu.cn/terms-and-conditions) on Wiley Online Library for rules of use; OA articles are governed by the applicable Creative Commons License

B4.6 JUICES AND FRUITS

Do you regularly drink juices or citric fruits (e.g., lemon, orange, grapefruit)?

No

Yes

Quit

If yes, what is your approximate intake (glasses/day)? __________

B4.7 CAFFEINATE

Do you regularly drink coffee, tea, or other caffeine beverages?

No

Yes

Quit

If yes, what is) your approximate intake (cups/day)? __________

13652842, 2023, 0, Downloaded from https://onlinelibrary-wiley-com-443.webvpn.bjmu.edu.cn/doi/10.1111/joor.13411. By Peking University Health, Wiley Online Library on [25/11/2023]. See the Terms and Conditions (https://onlinelibrary-wiley-com-443.webvpn.bjmu.edu.cn/terms-and-conditions) on Wiley Online Library for rules of use; OA articles are governed by the applicable Creative Commons License

B5. Additional factors assessment

B5.1 FAMILIAR BRUXISM SCREENING
Do you know of anyone in your family (for example, father, mother, children) who has had any history of bruxism occurrence?
No
Yes Father/Mother/Son/Daughter/Grandfather/Grandmother
Don't know

B5.2 FAMILIAR TOOTH WEAR SCREENING
Do you know of anyone in your family (for example, father, mother, children) who has tooth wear?
No
Yes Father/Mother/Son/Daughter/Grandfather/Grandmother
Don't know

B5.3 FAMILIAR OSA SCREENING
Do you know of anyone in your family (for example, father, mother, children) who has sleep apnea?
No
Yes Father/Mother/Son/Daughter/Grandfather/Grandmother
Don't know

B5.4 FAMILIAR OROFACIAL PAIN SCREENING
Do you know of anyone in your family (for example, father, mother, children) who has non-dental facial pain?
No
Yes Father/Mother/Son/Daughter/Grandfather/Grandmother
Don't know

B5.5 FAMILIAR GERD SCREENING
Do you know of anyone in your family (for example, father, mother, children) who has gastroaesophageal reflux disease?
No
Yes Father/Mother/Son/Daughter/Grandfather/Grandmother
Don't know

13652842, 2023, 0, Downloaded from https://onlinelibrary-wiley-com-443.webvpn.bjmu.edu.cn/doi/10.1111/joor.13411. By Peking University Health, Wiley Online Library on [25/11/2023]. See the Terms and Conditions (https://onlinelibrary-wiley-com-443.webvpn.bjmu.edu.cn/terms-and-conditions) on Wiley Online Library for rules of use; OA articles are governed by the applicable Creative Commons License
